# Azole Resistance in *Candida parapsilosis* From Patients With Burns in Mexico: A Genomic and Phylogenetic Analysis

**DOI:** 10.1111/myc.70161

**Published:** 2026-03-07

**Authors:** María de Lourdes García‐Hernández, Luis Ángel Nuñez‐García, Jossue Ortíz‐Álvarez, Marco Antonio Delaye‐Martínez, Marlen Flores‐Huacuja, María del Rocío Vazquez‐Olivares, Luis Fernando Espinosa‐Camacho, Gabriela Delgado‐Sapien, María del Rosario Morales‐Espinosa, Noé Becerra‐Lobato, Rafael Franco‐Cendejas, Elvira Garza‐González, Luis Ostrosky‐Zeichner, Tatiana Chávez‐Heres, Melissa Hernández‐Durán, Luis Esaú López‐Jácome, Claudia Adriana Colín‐Castro

**Affiliations:** ^1^ Laboratorio de Microbiología Clínica. Instituto Nacional de Rehabilitación Luis Guillermo Ibarra Ibarra Mexico Mexico; ^2^ Departamento de Bioquímica y Medicina Molecular. Facultad de Medicina Monterrey Nuevo León Mexico; ^3^ Programa “Investigadoras e Investigadores Por México”. Secretaría de Ciencia, Humanidades, Tecnología e Innovación (SECIHTI) Mexico Mexico; ^4^ Plan de Estudios Combinados en Medicina (MD‐PhD Program), Facultad de Medicina, Universidad Nacional Autónoma de México Mexico Mexico; ^5^ Centro Nacional de Referencia de Inocuidad y Bioseguridad Agroalimentaria, Servicio Nacional de Sanidad, Inocuidad y Calidad Agroalimentaria (SENASICA) Tecámac Mexico; ^6^ Departamento de Microbiología y Parasitología, Facultad de Medicina Universidad Nacional Autónoma de México Mexico City Mexico; ^7^ Subdirección de Investigación Biomédica Instituto Nacional de Rehabilitación Luis Guillermo Ibarra Ibarra Mexico City Mexico; ^8^ Division of Infectious Diseases, University of Texas Health Science Center Houston Texas USA; ^9^ Unidad de Vigilancia Epidemiológica Hospitalaria. Instituto Nacional de Rehabilitación Luis Guillermo Ibarra Ibarra Mexico City Mexico; ^10^ Departamento de Biología Facultad de Química. Universidad Nacional Autónoma de México Mexico City Mexico

**Keywords:** azole resistance, *Candida parapsilosis*, ERG11, patients with burns

## Abstract

**Background:**

*Candida* species are common causes of nosocomial infections. *Candida parapsilosis* has emerged as an important pathogen*.* Mortality rates associated with invasive *C. parapsilosis* infections range from 14.5% to 47%, with azole resistance estimated between 4% and 15%. Resistance mechanisms include mutations in *ERG11* that diminish azole binding, upregulation of ergosterol biosynthesis genes due to mutations in regulatory factors like *UPC2* and *NDT80*, and increased efflux pump activity.

**Methods:**

Thirty clinical strains of the *C. parapsilosis* complex were isolated from bloodstream cultures, biopsies and catheter tips from hospitalised patients from a major rehabilitation centre in Mexico between June 2011 and August 2016. Minimum inhibitory concentrations for fluconazole, voriconazole, itraconazole, caspofungin, micafungin, anidulafungin and amphotericin B were determined using the broth microdilution method. Whole genome sequencing was conducted using Illumina technology, with subsequent assembly and annotation. Analyses included antifungal resistance variant screening, SNP distance, average nucleotide identity and phylogenetic comparisons with publicly available genomes.

**Results:**

Most strains (96.7%, 29/30) were identified as *C. parapsilosis* sensu stricto, predominantly isolated from blood. Thirty‐three percent of isolates showed phenotypic resistance to fluconazole. The SNP‐distance and maximum‐likelihood phylogenetic analyses revealed two distinct haplotypes and the genotype 1: *ERG11* ^WT/Y132F^, *CDR1* ^I1287V/I1287V^ and *UPC2* ^WT/N455D^ was the most represented (8/10 isolates).

**Conclusions:**

*C. parapsilosis* sensu stricto was the predominant species isolated, with a notable proportion exhibiting resistance to FLU and VOR. The Y132F variant in the *ERG11* gene was present in most sequenced isolates.

## 1 Introduction

*Candida* species and yeast‐like (taxonomically reclassified into new genera and previously belonging to the genus *Candida*) are common causes of nosocomial infections. *Candida albicans* has been recognised as the most frequent species associated with infections; however, a shift towards higher frequency for non‐*albicans* species has been documented [1]. *Candida parapsilosis* has emerged as a predominant species in various settings, becoming one of the most identified non‐albicans species [2]. This yeast is a component of the human skin microbiota and can function as an opportunistic pathogen in immunocompromised individuals [1]. *C. parapsilosis* is part of a complex integrated by three cryptic species: *C. orthopsilosis, C. metapsilosis* and *C. parapsilosis* sensu stricto [3].

Mortality rates associated with invasive infections by *C. parapsilosis* range from 14.5% to 47% globally [2] and azole resistance within the complex is estimated between 4% [4] to 15% [5]. However, data on azole resistance in Mexico remain scarce. One study that included clinical isolates collected from 1999 to 2010 reported fluconazole resistance of 4.5% in *C. parapsilosis* sensu stricto isolates, with all *C. orthopsilosis* and *C. metapsilosis* isolates remaining susceptible [6]. In contrast, another study based on isolates collected from 2014 to 2016 documented fluconazole resistance of 54% in *C. parapsilosis* isolates [7].

Given the increasing concern regarding antifungal resistance and the limited availability of local surveillance data, this study aimed to identify mutations associated with azole resistance in isolates belonging to *C. parapsilosis* complex obtained from patients with burns treated at a referral hospital in Mexico City. Additionally, the study sought to contribute to the understanding of the molecular mechanisms underlying resistance in this clinically significant fungal species and to provide baseline information to support effective infection control and treatment strategies.

## 2 Material and Methods

### 2.1 Study Site, Culture Collection, Clinical Data and Statistical Analysis

This study was carried out at the Instituto Nacional de Rehabilitación Luis Guillermo Ibarra Ibarra (INR‐LGII), a centre specialising in rehabilitation and burn care.

For this study, 30 clinical strains of *C. parapsilosis* complex recovered from bloodstream cultures, biopsies and catheter tips from hospitalised patients from June 2011 to August 2016 were included. Isolates were identified with Vitek 2 compact (Biomérieux. Marcy‐l'Étoile, France) and were kept at −70°C in 20% glycerol Brain Heart Infusion.

For this study, clinical isolates were thawed, and identification was confirmed by Vitek MS (bioMérieux, Marcy‐l'Étoile, France). Clinical information from patients was retrieved from medical electronic records. Descriptive analysis using frequency measures and percentages was employed for categorical variables; median and interquartile range (IQR) were used for continuous variables.

This study was reviewed and approved by the local research committee under approval number 16/23.

### 2.2 Susceptibility Testing

Susceptibility to fluconazole (FLU), voriconazole (VOR), itraconazole (ITC), caspofungin (CAS), micafungin (MCF), anidulafungin (AND) and amphotericin B (AMB) was evaluated with the broth microdilution method and interpreted according to recommendations of CLSI M27‐A3 [8] to determine the minimum inhibitory concentration (MIC) for each antifungal; in addition, MIC_50_ and MIC_90_ were obtained, break points were considered according to CLSI M27M44S [9] and M57S [10]. For ITR and AMB, strains were defined as wild‐type (WT) and non‐wild‐type (nWT) categories according to the CLSI M57S guideline. *C. parapsilosis* ATCC 22019 and *Pichia kudriavzevii* (formerly *Candida krusei* ) ATCC 6528 were used as controls.

### 2.3 Whole Genome Sequencing

Isolates that showed resistance to at least one azole were selected for whole genome sequencing, with the aim of identifying the possible resistance mechanisms. Strains were grown on SDA and incubated at 30°C for 24 h. DNA extraction was performed using the Wizard Genomic DNA Purification Kit (Promega, Madison, Wisconsin, USA). Colonies were resuspended in 700 μL of Nucleic Lysis Solution and adjusted to a turbidity of 2 in the McFarland Scale. 10 μL of Proteinase K (10 mg/mL) and Lysozyme (10 mg/mL) was added and incubated at 37°C for 30 min; instructions from the manufacturer were followed after digestion. DNA quality was evaluated with NanoDrop One (Thermo Fisher Scientific, Waltham, Massachusetts, USA) and Qubit 4.0 fluorometer (Invitrogen, Waltham, Massachusetts, USA). Library preparation was performed with the Illumina DNA Prep Kit (Illuminam, San Diego, California, USA) and consisted of a tagging step, posttagmentation cleanup, library amplification and indexing IDT for Illumina DNA/RNA UD Indexes (Illumina, San Diego, California, USA). The libraries were then purified by a washing step with fresh 80% ethanol and then resuspended with the RSB buffer included in the kit. Subsequently, library quality assessment was performed with the 4200 TapeStation System (Agilent, Santa Clara, California, USA) and the Qubit 4.0 fluorometer (Invitrogen, Waltham, Massachusetts, USA). The libraries were normalised to 4 nM and two pools were made. The first pool was sequenced using a NextSeq High Output Flow Cell V2.5 and an NSQ 500Hi‐Output Reagment V2 cartridge (300 cycles) on the Illumina NextSeq 550 platform. The second pool was sequenced using a Flow cell V2 and a V2 cartridge (300 cycles) on the Illumina MiSeq platform, both in a paired‐end configuration. The samples were sequenced in the Sequencing Laboratory of the Centro Nacional de Referencia de Inocuidad y Bioseguridad Agroalimentaria (SENASICA).

### 2.4 Genome Assembly and Annotation

Raw sequencing reads were processed using Trim_Galore v0.6.10 [11] to remove low‐quality bases and adapters. *De novo* assembly was performed using three strategies: standalone SPAdes v4.2.0, Shovill‐SPAdes and Shovill‐SKESA [12, 13]. Metrics for assessing assembly quality were determined with QUAST [14]; the assembly with the fewest content of contigs was selected for further processing. Genome completeness and contamination were evaluated using BUSCO [15] with the *Saccharomycetes* dataset. Mitochondrial DNA contigs were manually removed using Seqkit [16].

### 2.5 Antifungal Resistance Variants Screening

Trimmed reads were aligned to the *C. parapsilosis* CDC317 reference genome (NCBI accession GCF_000182765.1) using BWA‐MEM [17]. SAM‐formatted alignments were converted to BAM format using Samtools v1.20 [18]. Variants were identified using BCFtools v1.20: a pileup was generated, followed by variant calling with the BCFtools ‘call’ function [18]. Variant annotation was performed using SnpEff v5.2 [19]. Sequences for genes with reported mutations associated with antifungal resistance (Table S1) were extracted from the *Candida* genome database: *CAP1, CDR1, CPH1, ERG11, FCR1, MCM1, MDR1, MRR1, NDT80, REP1, STB5, TAC1 and UPC1* [20, 21, 22, 23, 24, 25, 26, 27, 28, 29, 30]. Annotations (GFF format) for the *C. parapsilosis* CDC317 reference genome were obtained from NCBI [31], and gene features corresponding to resistance‐associated *loci* were extracted. We compared the annotated variant file to these extracted properties using BEDTools v2.31.1 [32] ‘intersect’ function to identify variants overlapping these features.

### 2.6 SNP‐Distance, Average Nucleotide Identity and Phylogenetic Analysis

Taxonomy was verified by calculating Average Nucleotide Identity (ANI) using ANIclustermap [33] between isolates and reference genomes of *Candida* species and closely related yeasts. ANI values > 95% were considered as indicative of the same species [34]. For phylogenetic analysis, assemblies were mapped to the *C. parapsilosis* CDC317 reference genome using Snippy [35]. The whole‐genome alignment was processed with Genealogies Unibased By recomBinations in Nucleotide Sequences (Gubbins) [36] to mask recombination sites and obtain the core‐genome SNPs alignment; SNP‐distances were calculated using snp‐dists [37]. The core alignment was used to construct a maximum‐likelihood (ML) phylogenetic tree with IQ‐TREE2 [38], the best‐fit substitution model was determined by the ModelFinder module, and branch support was estimated from 1000 ultrafast bootstrap replicates.

### 2.7 Comparison With Genomic Public Records

*C. parapsilosis* sensu stricto genome assemblies were retrieved from the NCBI database. Duplicate assemblies of the CDC317 were removed. Both the *de novo* assemblies and the extracted genomes were analysed using the strategy for phylogenetic analysis described above. Additionally, the final tree and SNP‐distances were used to determine population structure using rhierbaps [36].

### 2.8 Data Availability Statement

Raw sequencing reads and assembled genomes were deposited in the National Center for Biotechnology Information (NCBI) under project number PRJNA1287628.

## 3 Results

### 3.1 Identification and Clinical Features of Patients

Clinical strains were recovered from 28 patients with burns. Most strains were isolated from bloodstream cultures (13/30; 43.3%), followed by biopsies (12/30, 40%) and catheter tips (5/30, 16.7%). Twenty‐nine strains were identified as *Candida parapsilosis sensu stricto* (96.7%) and one as *Candida orthopsilosis* (3.3%).

Most patients were male (82%) with a median age of 36 years. The extent of burns involved approximately 45% (30–58) of the Total Surface Body Area (TSBA). Almost 75% (22/28) exhibited third‐degree burns (Table 1).

**TABLE 1:** Sociodemographic and clinical characteristics of patients.

Clinical characteristics	Overall (*n* = 28)
Sex (male)	23 (82)
Age (years)^a^	36 (20–54)
Comorbidities	
Diabetes mellitus *n* (%)	2 (7.1)
Hypertension *n* (%)	5 (18)
Obesity *n* (%)	5 (18)
Burn injury mechanism	
Fire *n* (%)	21 (75)
Scald *n* (%)	1 (3.6)
Electric *n* (%)	4 (14)
Mixed: fire + electricity *n* (%)	2 (7.1)
Burn severity	
2nd degree *n* (%)	7 (25)
3rd degree *n* (%)	21 (75)
Burn Total Surface Body Area^a^	45 (30–58)
ABSI score^a^	8 (6–10)

Abbreviation: ABSI, Abbreviated Burn Severity Index.

^a^ Median (IQR: Interquartile Range).

Bacterial coinfection was present in 54% of the patients, with *Pseudomonas aeruginosa* (25%; 7/28) being the most frequent, followed by *Acinetobacter baumannii* (21.42%; 6/28). Three patients showed coinfection with other yeast species; one with *C. albicans* , one with *C. tropicalis* and one with *Pichia kudriavzevii* and *C. albicans*.

Antimicrobial treatment was adjusted to cover both invasive candidiasis and bacterial infections. With respect to the antifungal therapy, FLU and VOR were the main prescribed antifungals, followed by echinocandins (AND, CAS) or AMB, with a median duration of 14 days (IQR 7–19) (Table 2). In 10 (33.3%) patients, azole resistance was detected. These patients received FLU (*n* = 6), AND (*n* = 3) and AMB (*n* = 1). Clinical evolution was favourable in all patients, with the exception of one patient, who died 6 days after invasive candidiasis was detected. This patient received FLU treatment and had a burn TSBA of 50%, with concomitant coinfection in soft tissues with *P. aeruginosa* and *A. baumannii*. On the other hand, in the azole‐susceptible *C. parapsilosis* group, three patients died during the treatment period. All of these patients had burn injuries between 50% and 60% of TSBA, with polymicrobial infections both in the burn injuries and bloodstream. No adverse events were reported during the treatment duration. These results are shown in Tables [Link], [Link].

**TABLE 2:** Treatment features and clinical outcomes of the patients.

Features	Overall (*n* = 28)
Yeast‐associated infection	
Invasive bloodstream infection *n* (%)	12 (43%)
Catheter tip infection *n* (%)	4 (14%)
Soft tissue infection *n* (%)	12 (43%)
Anti‐fungal therapy	
FLU *n* (%)	19 (68%)
VOR *n* (%)	1 (3.6%)
AND *n* (%)	3 (11%)
CAS *n* (%)	3 (7.1%)
AMB *n* (%)	2 (7.1%)
Bacterial coinfection *n* (%)	15 (54%)
Treatment duration (days)^a^	14 (7–19)
Length of stay (LOS) (days)^a^	63 (34–83)
Overall mortality *n* (%)	4 (14%)

Abbreviations: AMB, amphotericin B; AND, anidulafungin; CAS, caspofungin; FLU, fluconazole; VOR, voriconazole.

^a^ Median (IQR, Interquartile Range).

### 3.2 Susceptibility to Azoles in Clinical Strains of *C. parapsilosis* Complex Isolated From Burned Patients

Approximately 30% of isolates were resistant to FLU (MIC 16–64 μg/mL) and VOR (MIC 1–16 μg/mL); meanwhile, six strains (16.6%) were nWT to ITR, and 10/30 isolates were nWT for AMB (Table 3). All isolates were susceptible to the antifungals CAS, MCF and AND. The MIC_90_ value for the antifungals ITR and AND is 1 μg/mL; for VOR, CAS, MCF and AMB, it is 2 μg/mL; and for FLU, it is 32 μg/mL (Table 3).

**TABLE 3:** Minimum inhibitory concentrations (MIC) ranges and categorical susceptibility results.

Antifungal	Minimum inhibitory concentrations (MIC) (μg/mL)			Categorical susceptibility *n* (%)			Epidemiological definition *n* (%)	
	Range (μg/mL)	MIC_50_	MIC_90_ (μg/mL)	S	SDD/I	R	WT	nWT
FLU	0.03–64	1	32	19 (63.3)	1 (3.3)	10 (33.3)	—	—
VOR	0.03–16	0.03	2	20 (66.6)	1 (3.3)	9 (30)	—	—
ITR^a^	0.03–16	0.25	1	—	—	—	25 (83.3)	5 (16.6)
CAS	0.125–2	1	2	30 (100)	0	0	—	—
MCF	0.125–4	1	2	29 (96.6)	1 (3.3)	0	—	—
AND	0.03–2	1	1	30 (100)	0	0	—	—
AMB^a^	0.5–2	1	2	—	—	—	20 (66.6)	10 (33.3)

Abbreviations: AMB, amphotericin B; AND, anidulafungin; CAS, caspofungin; FLU, fluconazole; I, intermediate; ITR, itraconazole; MCF, micafungin; MIC, Minimal inhibitory concentration; nWT, non‐wild‐type; R, resistant; S, susceptible; SDD, susceptibility dose‐dependent; VOR, voriconazole; WT, wild‐type.

^a^ Without clinical breakpoints.

### 3.3 Phylogenetic Relationships and Antifungal Resistance‐Associated Mutations

Sequenced isolates were identified as part of the *C. parapsilosis* complex since all genomes lie within the 95% ANI species threshold (Figure S1). Phylogenomic analysis showed clustering of 9/10 sequenced isolates in this study, which formed a monophyletic clade (Figure 2). The closest related genomes from Genbank were isolates collected in the United States between 2014 and 2017 and in Australia during 2021 and 2022. These isolates clustered closely together, with fewer than 200 SNP differences among them (Figure S2). On the other hand, isolate H029 was phylogenetically distant from the other sequenced isolates, showing approximately 2400 SNPs differences and clustered with strains predominantly isolated in China, mostly from 2019, suggesting it represents a distinct lineage.

**FIGURE 1:**
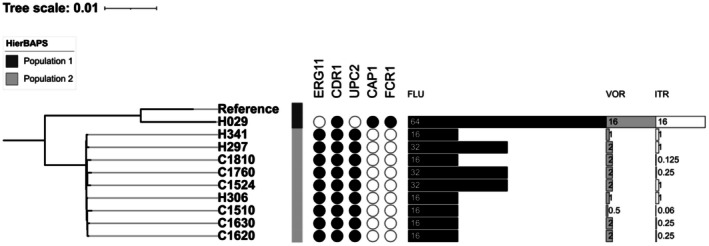
Midpoint‐rooted maximum likelihood phylogenetic tree based on SNP distances of the sequenced isolates. Genes associated with antifungal‐resistance are shown on the right binary data representation. Bar‐chart values depict minimum inhibitory concentrations for fluconazole (FLU), voriconazole (VOR) and itraconazole (ITR). Numbers in bars represent values for minimum inhibitory concentrations.

Strain H029 had a unique mutational profile characterised by variants in *CDR1*, *CAP1* and *FCR1* (Figure 1). Conversely, the primary clade shared mutations in *ERG11*, *CDR1* and *UPC2*. The genotype 1: *ERG11* ^WT/Y132F^, *CDR1* ^I1287V/I1287V^ and *UPC2* ^WT/N455D^, was present in 8/10 isolates. A closely related genotype 2: *ERG11* ^Y132F/Y132F^, *CDR1* ^I1287V/I1287V^ and *UPC2* ^WT/N455D^, differed by carrying a homozygous Y132F mutation in *ERG11*, and occurred in isolate C1810 (Table 4). Missense variants in these genotypes have a putative moderate impact according to SnpEff annotation. The more divergent genotype 3: *CDR1* ^WT/I1287V^, *CAP1* ^WT/C478*^ and *FCR1* ^Q211*/Q211*^ was identified in isolate H029. The nonsense mutations in *CAP1* and *FCR1* are predicted to have a putative high impact according to SnpEff. All the identified variants have a sequencing depth > 77, ensuring confidence in the variant calling.

**TABLE 4:** MIC of azole‐resistant strains and mutations found in *ERG11*, *CDR1*, *UPC2*, *CAP1* and *FCR1* genes.

Strain	Clinical specimen	Species	MIC (μg/mL)			Genotype				
			FLU	VOR	ITR	*ERG11*	*CDR1*	*UPC2*	*CAP1*	*FCR1*
H029	Blood	*C. parapsilosis*	**64**	**16**	**16**	—	WT/I1287V	—	WT/C478*	Q211*/Q211*
H297	Blood	*C. parapsilosis*	**32**	**2**	**1**	WT/Y132F	I1287V/I1287V	WT/N455D	—	—
H306	Blood	*C. parapsilosis*	**16**	**1**	**1**	WT/Y132F	I1287V/I1287V	WT/N455D	—	—
H341	Blood	*C. parapsilosis*	**16**	**1**	**1**	WT/Y132F	I1287V/I1287V	WT/N455D	—	—
C1510	Biopsy	*C. parapsilosis*	**16**	0.5	0.06	WT/Y132F	I1287V/I1287V	WT/N455D	—	—
C1524	Biopsy	*C. parapsilosis*	**32**	**2**	**1**	WT/Y132F	I1287V/I1287V	WT/N455D	—	—
C1620	Biopsy	*C. parapsilosis*	**16**	**2**	0.25	WT/Y132F	I1287V/I1287V	WT/N455D	—	—
C1630	Biopsy	*C. parapsilosis*	**16**	**2**	0.25	WT/Y132F	I1287V/I1287V	WT/N455D	—	—
C1760	Biopsy	*C. parapsilosis*	**32**	**2**	0.25	WT/Y132F	I1287V/I1287V	WT/N455D	—	—
C1810	Biopsy	*C. parapsilosis*	**16**	**2**	0.125	Y132F/Y132F	I1287V/I1287V	WT/N455D	—	—

*Note:* MICs that correspond to resistance are highlighted in bold.

Abbreviations: FLU, Fluconazole; ITR, Itraconazole.

All strains with genotype 1 exhibited resistance to FLU; seven were resistant and one SDD to VOR For ITR, four showed a WT phenotype. The strain with genotype 2 (C1810) showed similar MICs, resistance to FLU and VOR and WT for ITR. In contrast, strain H029 (genotype 3) was resistant to all azoles, with the highest MICs evaluated (Table 4).

### 3.4 Phylogenetic Relationship With Public Genomes

**FIGURE 2:**
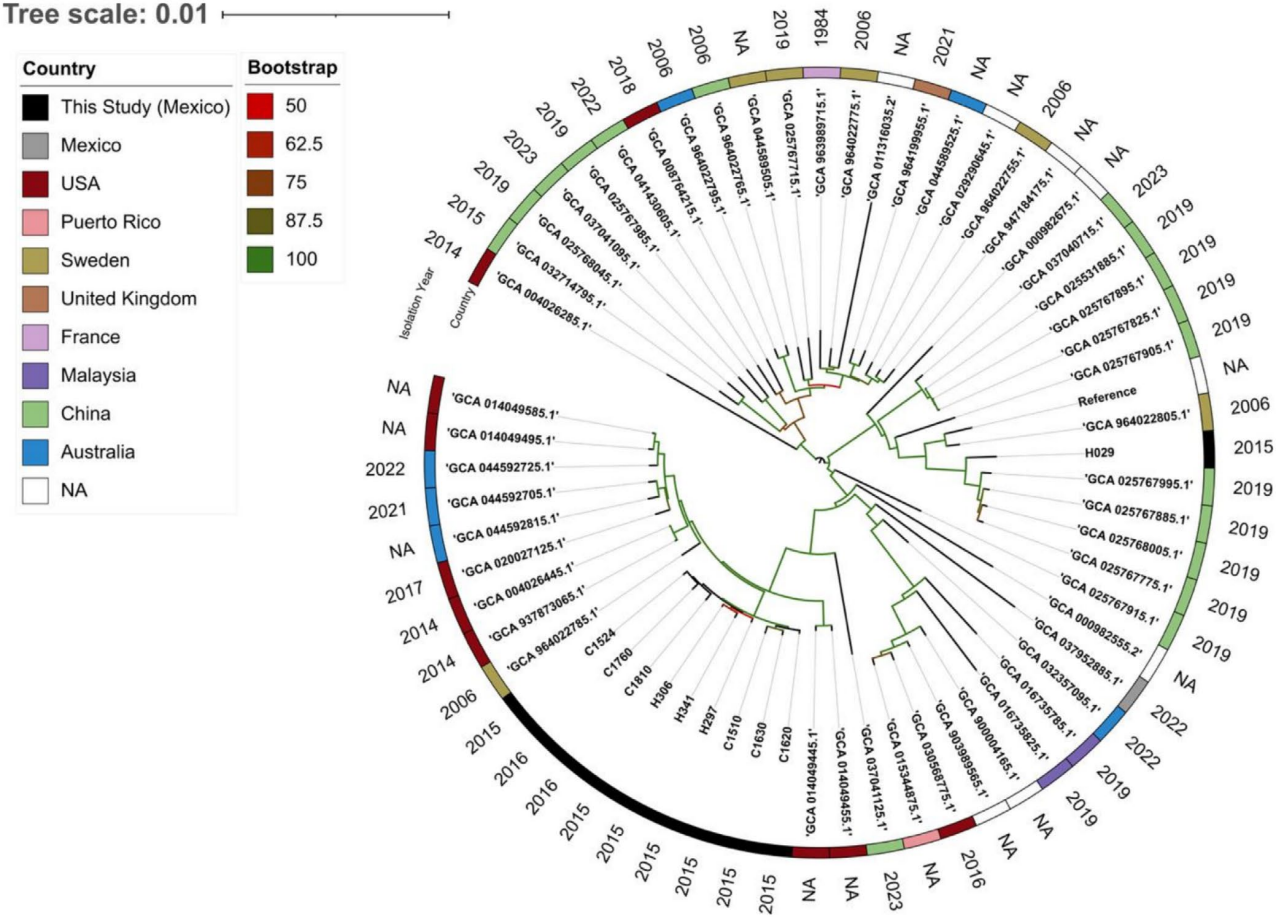
Midpoint‐rooted maximum likelihood phylogenetic tree based on SNP distances. Publicly available *Candida parapsilosis* genomes from the NCBI RefSeq database (*n* = 55) and the sequence isolates were compared. ‘N/A’ indicates unavailable metadata. The outermost annotation layer represents the year of isolation.

A total of 53 unique assemblies were obtained. A strong phylogenetic relationship was observed among 9/10 sequenced isolates in this study, which clustered together within a distinct clade (Figure 2). The closest related genomes in the NCBI Assembly database were isolates collected in the United States between 2014 and 2017 and in Australia during 2021 and 2022; all were classified as belonging to population one according to hierBAPS analysis. The remaining isolate, H029, was phylogenetically distant from the others and clustered with strains predominantly isolated in China, mostly from 2019.

To the best of our knowledge, this is the first study that described whole genome sequencing data from *C. parapsilosis* sensu stricto from strains isolated within a clinical context in Mexico.

## 4 Discussion

Patients with burns are highly susceptible to nosocomial infections due to the compromise of primary mucosal and skin barriers, and yeast plays a significant role, resulting in approximately 180,000 deaths each year [39]. Thus, in this study, we aimed to identify mutations associated with azole resistance in *C. parapsilosis* isolates obtained from burn patients treated at a specialised hospital in Mexico.

Invasive fungal infections in burned patients are predominantly caused by *Candida* species, particularly *C. albicans* and *C. parapsilosis* [40, 41, 42]. Within the *C. parapsilosis* complex, *C. parapsilosis* sensu stricto is the most reported species, with prevalence rates ranging from 88.5% to 95.3% [43, 44]. In contrast, *C. orthopsilosis* and *C. metapsilosis* account for 3.1%–8.4% and 1.6%–4.2% of isolates respectively [6, 45, 46]. Consistent with these reports, *C. parapsilosis* sensu stricto represented 96.7% of the isolates obtained from patients with burns in our study.

Invasive candidiasis has a global incidence rate reported in 12.1 cases/100,000 population, and is associated with mortality rates between 30% and 90% [43, 44, 47].

A recent ICU‐based study from Iran (2021–2023), including 353 burn patients reported yeast bloodstream infection in 15.3% of cases. In that cohort, mortality was significantly associated with advanced age, broad‐spectrum antibiotic exposure, yeast bloodstream infection, extensive TBSA involvement (> 50%) and prolonged LOS [48]. In our study, these same risk factors were prevalent: nearly half of the patients received broad‐spectrum antibiotics, 43% developed bloodstream infection, 45% had extended TSBA involvement and the median LOS was 63 days. In the same study, resistance to fluconazole and voriconazole was reported in 28% and 18.2% of isolates respectively [48].

Although azole resistance in invasive candidiasis has been associated with higher mortality, its relation with antifungal treatment failure in the burn patient might be difficult to elucidate. In this setting, mortality is often driven by the severity of the burn injury itself and by concurrent infections, most of them with multidrug‐resistant profiles. In our study, overall mortality was 14.3%, and all deaths occurred in patients with > 50% TBSA as well as concomitant bacterial bloodstream infection. Only one fatal case involving an azole‐resistant isolate was observed, and death was difficult to attribute to the azole's resistance.

Worldwide, azole resistance in *C. parapsilosis* is reported to be below 15% [4, 5]. In contrast, our study detected resistance percentages of 32.3% for FLU, 29% for VOR and 16.1% for ITR. When compared to two Mexican studies, our findings show intermediate resistance levels. Treviño et al. reported a much lower fluconazole resistance rate of 4.5%, although 56.6% of their isolates were obtained from blood cultures and the clinical origin of the remaining isolates was not specified [6]. Conversely, our resistance rates were lower than those reported by Corzo et al. who found a fluconazole resistance rate of 52% in blood‐derived clinical isolates [7]. Similar to our study, both previous reports were based on single‐centre samples, overlapping with the time frame of Corzo et al. (2011–2016) [7].

Atalay et al. identified intra‐abdominal surgery, hypoalbuminaemia and previous use of FLU as risk factors for developing azole‐resistant bloodstream infection by *C. parapsilosis* [49]. Similarly, Semet et al. reported the presence of a central venous catheter, colostomy and age over 66 years as risk factors [50]. In our population, 43% of patients developed catheter‐associated bloodstream infection, and 68% were treated with FLU, factors that may have contributed to the emergence of azole‐resistant infections in our cohort. However, detailed data on topical antifungal or antibacterial treatments, including wound irrigation protocols, were unavailable. Consequently, potential associations between local treatments and azole resistance could not be formally assessed.

Studies such as those by Rodriguez et al. [51] have demonstrated significant genetic diversity among clinical isolates. The work is focused on the amplification of eight polymorphic microsatellite regions by PCR [51, 52]. However, other works have reported a limited variability at the DNA level. For instance, Tavanti et al. used Amplification Fragment Length Polymorphism (AFLP) analysis on 62 clinical strains from different geographic regions and found reduced genetic diversity [53]. This finding is consistent with the work of Dr. Valach, who observed low genetic variability in *C. parapsilosis* compared to *C. metapsilosis* and *C. orthopsilosis* based on mitochondrial genome analysis [54]. These studies support the hypothesis that *C. parapsilosis* may have a recent evolutionary origin. This context is relevant to our findings, in which most of the sequenced azole‐resistant strains (9/10) belonged to the same population and centre. In burn patients specifically, resistance rates to FLU and VOR were 28% and 18.2% respectively. For *C. parapsilosis* , resistance rates are reported at 20% for FLU and 8% for VOR [48].

The *ERG11* Y132F mutation is associated with reduced susceptibility to azole antifungals across *Candida* species [7, 55, 56]. In this study, nine out of 10 isolates harboured at least one allele carrying this mutation. No clear correlation was observed between *ERG11* Y132F zygosity and azole resistance phenotypes, as isolate C1810 carries the *ERG11* ^Y132F/Y132F^ genotype but exhibits lower MICs when compared to strains heterozygous for this mutation. These findings suggest that additional factors not explored in this study, such as polymorphisms in other transcriptional factor genes, differences in virulence factor content (particularly biofilm formation) [57], epigenetic regulation or copy number variations and complex chromosomal rearrangements [26], may contribute to the observed resistance patterns.

Mutations were also detected in *CDR1* and *UPC2*, both genes known to be associated with antifungal resistance [58]. Two studies reported the I1287V mutation in *CDR1* [59, 60]; however, only one has linked this mutation to increased MICs for antifungals [59]. Different studies have analysed the relevance of mutations in *UPC2* for the overexpression of ergosterol biosynthesis genes [61, 62, 63], which contributes to azole nonsusceptibility [58]. Although numerous *UPC2* mutations have been described [64, 65, 66], the functional impact of the N455D substitution identified in this study has not been elucidated. Notably, this substitution is located within the zinc‐finger domain required for transcription factor activity, which may suggest a potential effect on the regulation of gene expression [67].

Mutations with a high predicted impact on protein function were identified in strain H029, which also exhibited the highest MICs across all tested antifungal agents and notably lacked the common *ERG11* Y132F mutation. Loss‐of‐function mutations in *FCR1*, a negative regulator of the *CDR1* efflux pump, might be associated with FLU resistance in *C. albicans* [25, 68]. Isolate H029 harboured a homozygous loss‐of‐function mutation in this gene, suggesting a constitutive upregulation of *CDR1* and leading to azole resistance. In contrast, *CAP1*, a positive regulator of the *MDR1* efflux pump [20], carried a heterozygous loss‐of‐function mutation, which could suggest reduced *MDR1* expression in this isolate, partially contradicting the hypothesis of efflux‐mediated resistance. However, *CAP1* is partially haplosufficient [69], at least in promoting virulence gene expression, which might suggest that the probable effect on *MDR1* under expression is less pronounced.

Phylogenetic and population structure analysis suggest that nine of the 10 sequenced isolates form a distinct lineage within our dataset, a finding supported by their convergent genotypes in antifungal resistance‐associated genes. These isolates were collected between 2015 and 2016, which, along with the low pairwise SNP‐distance, suggests clonal persistence during the observation period. In contrast, strain H029 was identified as a genotypically and phylogenetically distinct lineage with more than 2400 SNPs relative to all other sequenced isolates in this study. This divergence is further supported by the geographical clustering of strain H029 with isolates originating from China, whereas the remaining isolates clustered with strains from the United States and Australia. However, the temporal gap between H029 and the closest publicly available genomes, together with the absence of patient travel or referral history, limits inferences regarding its geographic origin or transmission route.

This study has limitations. First, it was conducted at a single centre, and therefore, the results do not represent the broader epidemiological landscape in Mexico. Second, the study population consisted exclusively of patients with burns, which may limit the generalisability of the findings to other types of infections or patient groups.

Third, the interpretation of the identified variants relied on previously reported data, and no functional validation or additional analyses were performed. Furthermore, only azole‐resistant isolates were sequenced; as a consequence, the generated data do not allow us to assess epidemiological factors that drive azole resistance. Consequently, the clinical and biological significance of the genotype observed in strain H029 remains speculative.

## 5 Conclusions

In this study, *C. parapsilosis* sensu stricto was the predominant species isolated from patients with burns at a specialised rehabilitation in Mexico, with a notable proportion exhibiting resistance to FLU and VOR. The Y132F variant in the *ERG11* gene was present in most sequenced isolates, which clustered phylogenetically with strains previously reported in the United States and Mexico.

Despite sharing lineage and genotypes, isolates with *ERG11* mutation expressed variable resistance to the antifungals that belong to the azoles, suggesting the contribution of other molecular mechanisms not explored in this study.

One phylogenetically distinct isolate, related to strains from China, exhibited elevated azole MICs in the absence of known resistance‐associated mutations, further supporting the need for comprehensive molecular analyses to elucidate alternative pathways of antifungal resistance. The narrowing of the phylogenetic spectrum of *C. parapsilosis* cannot be used to exclude the possibility of clonal distribution or the occurrence of outbreaks within our institution.

## Author Contributions

María de Lourdes García‐Hernández and Luis Ángel Nuñez‐García: conceptualisation, methodology, formal analysis, investigation, writing, original draft, writing, review and editing. Jossue Ortíz‐Álvarez and Marco Antonio Delaye‐Martínez: formal analysis, data curation, writing, original draft, writing, review and editing. Marlen Flores‐Huacuja, María del Rocío Vazquez‐Olivares, Luis Fernando Espinosa‐Camacho, Gabriela Delgado‐Sapien, María del Rosario Morales‐Espinosa, Tatiana Chávez‐Heres and Noé Becerra‐Lobato: formal analysis, data curation. Rafael Franco‐Cendejas and Elvira Garza‐González: formal analysis, writing, original draft, writing, review and editing. Luis Ostrosky‐Zeichner: original draft, writing, review and editing. Tatiana Chávez‐Heres: formal analysis. Luis Esaú López‐Jácome: conceptualisation, validation, supervision, project administration, investigation, writing, original draft, writing, review and editing. Claudia Adriana Colín‐Castro: conceptualisation, validation, investigation, writing, original draft, writing, review and editing.

## Funding

The authors have nothing to report.

## Conflicts of Interest

Pertinent to this work, LO has received research grants and/or consulting honoraria from the following companies: Scynexis, Melinta, GSK, Pulmocide, F2G, Basilea, Pfizer, Gilead, T2 Biosystems, Octapharma, Meiji, Stendhal, Knight and Eurofins Viracor. He is partially funded by the National Center for Advancing Translational Sciences (NCATS), National Institutes of Health, through UTHealth‐CCTS Grant Number UL1TR003167 and contract U01CK000692, Centers for Disease Control and Prevention. The contents of this publication are solely the responsibility of the authors and do not necessarily represent the official views of the NIH or CDC.

## Supporting information


**Table S1:**Genes with reported mutations associated with antifungal resistance.

**Table S2:**Sociodemographic characteristics of the cohort.

**Table S3:**Treatment characteristics and clinical outcomes between study groups.

**Figure S1:**Average Nucleotide Identity between the sequenced isolates and representative yeast species. Numbers in the boxes represent de ANI values (%).

**Figure S2:**Recombination‐masked SNP‐distance matrix of the sequenced isolates.

## Data Availability

The data that supports the findings of this study are available in the Supporting Information of this article.
