# Supplementary material for: Azole Resistance in Candida parapsilosis From Patients With Burns in Mexico: A Genomic and Phylogenetic Analysis
Source: Mycoses. 2026 Mar 7;69(3):e70161. doi: 10.1111/myc.70161 (PMC12966976; doi:10.1111/myc.70161)
Supplement: Supplementary file 1 — Table S1:Genes with reported mutations associated with antifungal resistance. [file MYC-69-e70161-s001.docx]

**Supplementary Table 1. Genes with reported mutations associated with antifungal resistance.**

| Gene | Function | Species with reported AFR association | Locus tag | Reference |
| --- | --- | --- | --- | --- |
| *CAP1* | MDR1 positive regulator | *C. alibcans* | CPAR2_405030 | [20] |
| *CDR1* | Efflux pump | *Candida* spp. | CPAR2_405290 | [21, 22] |
| *CPH1* | MDR1 negative regulator | *C. alibcans* | CPAR2_208600 | [23] |
| *ERG11* | Lanosterol demethylase | *Candida* spp. | CPAR2_303740 | [26] |
| *FCR1* | CDR1 negative regulator | *C. albicans* | CPAR2_13640 | [25] |
| *JJJ1* | CDR1 negative regulator | *C. glabrata* | CPAR2_802280 | [24] |
| *MDR1* | Efflux pump | *Candida* spp. | CPAR2_301760 | [26] |
| *MRR1* | MDR1 positive regulator | *Candida* spp. | CPAR2_807270 | [21, 22] |
| *NDT80* | CDR1 positive regulator | *C. albicans* | CPAR2_213640 | [27, 28] |
| *REP1* | MDR1 negative regulator | *C. alibcans* | CPAR2_800080 | [29] |
| *STB5* | CDR1 negative regulator | *C. glabrata* | CPAR2_109760 | [30] |
| *TAC1* | CDR1 positive regulator | *C. albicans* | CPAR2_303510 | [21, 22] |
| *UPC2* | *ERG* genes positive regulator | *C. albicans* | CPAR2_207280 | [26] |
