# Supplementary material for: Azole Resistance in Candida parapsilosis From Patients With Burns in Mexico: A Genomic and Phylogenetic Analysis
Source: Mycoses. 2026 Mar 7;69(3):e70161. doi: 10.1111/myc.70161 (PMC12966976; doi:10.1111/myc.70161)
Supplement: Supplementary file 2 — Table S2:Sociodemographic characteristics of the cohort. [file MYC-69-e70161-s002.docx]

**Supplementary** **Table 2**. Sociodemographic characteristics of the cohort.

|  | Azole susceptible group  (n=18) | Azole resistant group  (n=10) | p-Value^a^ |
| --- | --- | --- | --- |
| Sex (male) n (%) | 16 (89) | 7 (70) | 0.3 |
| Age (years) median (IQR) | 29 (19-39) | 54 (43-66) | **0.002** |
| Comorbidities   - Diabetes mellitus n (%) - Hypertension n (%) - Obesity n (%) | 0 (0)  2 (11)  4 (22) | 2 (20)  3 (30)  1 (10) | 0.12  0.3  0.6 |
| Burn injury mechanism   - Fire n (%) - Scald n (%) - Electric n (%) - Mixed: fire + electricity n (%) | 14 (78)  1 (5.6)  3 (17)  0 (0) | 7 (70)  0 (0)  1 (10)  2 (20) | 0.3 |
| Burn severity   - 2^nd^ grade n (%) - 3^rd^ grade n (%) | 6 (33)  12 (67) | 1 (10)  9 (90) | 0.4 |
| Burn Total Surface Body Area (%) | 43 (24-60) | 48 (35-56) | 0.4 |
| ABSI score median (IQR) | 7 (6-9) | 10 (9-10) | **0.001** |

1. Fisher exact test; Wilcoxon rank sum test; unpaired t-student.
