# Supplementary material for: Azole Resistance in Candida parapsilosis From Patients With Burns in Mexico: A Genomic and Phylogenetic Analysis
Source: Mycoses. 2026 Mar 7;69(3):e70161. doi: 10.1111/myc.70161 (PMC12966976; doi:10.1111/myc.70161)
Supplement: Supplementary file 3 — Table S3:Treatment characteristics and clinical outcomes between study groups. [file MYC-69-e70161-s004.docx]

**Supplementary Table 3.** Treatment characteristics and clinical outcomes between study groups.

|  | Azole susceptible group  (n=18) | Azole resistant group  (n=10) | p-Value^a^ |
| --- | --- | --- | --- |
| Infection positive culture   - Blood culture n (%) - Catheter tip n (%) - Biopsy n (%) | 8 (44)  4 (22)  6 (33) | 4 (40)  0 (0)  6 (60) | 0.3 |
| Anti-fungal therapy   - FLU n (%) - VOR n (%) - AND n (%) - CAS n (%) - AMB n (%) | 13 (72)  1 (5.6)  0 (0)  2 (11)  1 (5.6) | 6 (60)  0 (0)  3 (30)  0 (0)  1 (10) | 0.12 |
| Treatment duration (days) | 14 (9-15) | 14 (6-20) | >0.9 |
| Bacterial co-infection n (%) | 7 (39) | 8 (80) | 0.055 |
| Length of stay (LOS)(days) | 50 (30-69) | 90 (75-132) | **0.004** |
| Overall mortality | 3 (17) | 1 (10) | >0.9 |

1. Fisher exact test; Wilcoxon rank sum test.
