# Supplementary material for: Azole Resistance in Candida parapsilosis From Patients With Burns in Mexico: A Genomic and Phylogenetic Analysis
Source: Mycoses. 2026 Mar 7;69(3):e70161. doi: 10.1111/myc.70161 (PMC12966976; doi:10.1111/myc.70161)
Supplement: Supplementary file 4 — Figure S1:Average Nucleotide Identity between the sequenced isolates and representative yeast species. Numbers in the boxes represent de ANI values (%). [file MYC-69-e70161-s003.docx]

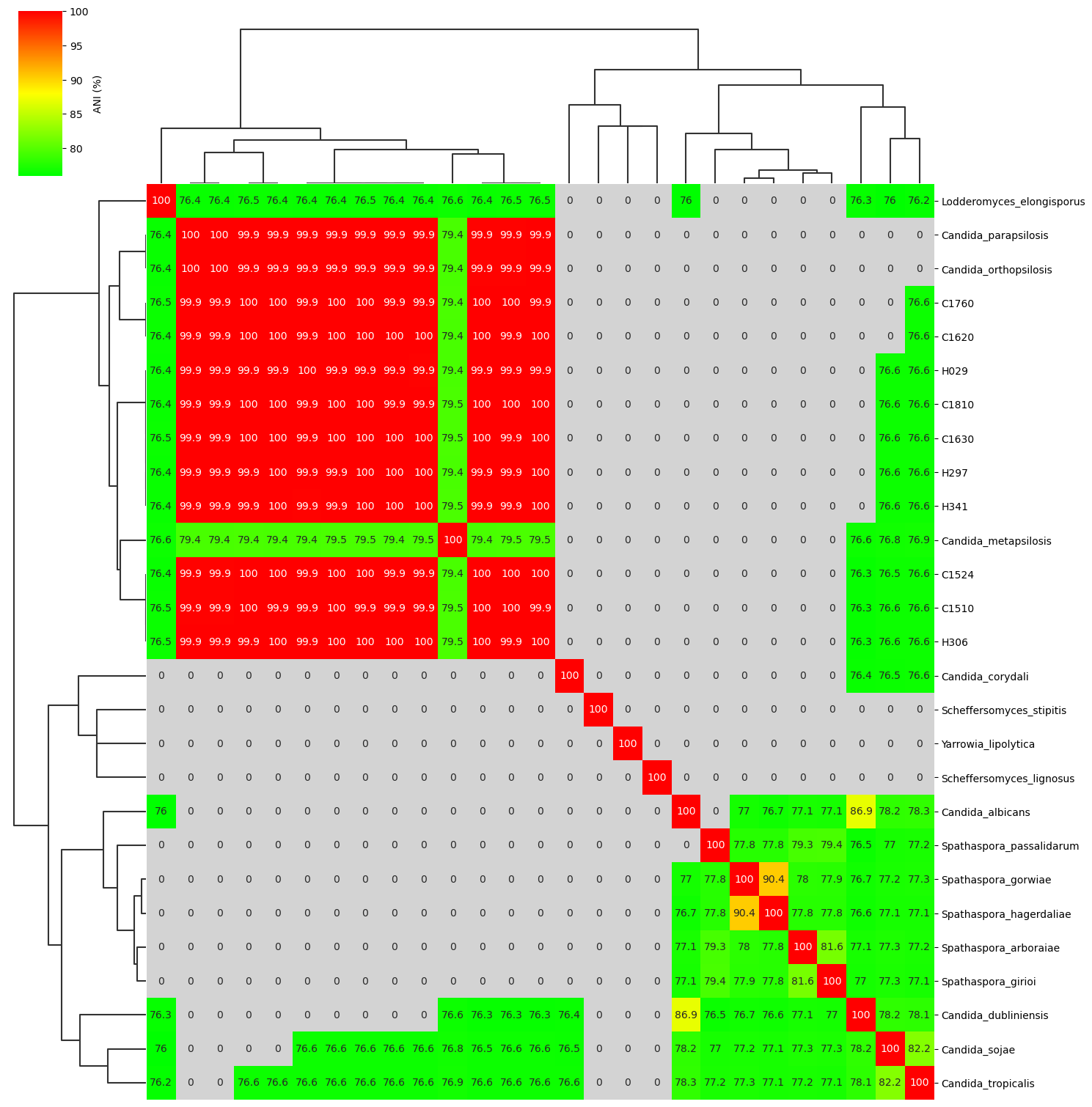


**Supplementary Figure 1.** Average Nucleotide Identity between the sequenced isolates and representative yeast species. Numbers into the boxes represent de ANI values (%).
