# Supplementary figures and images for: Azole Resistance in Candida parapsilosis From Patients With Burns in Mexico: A Genomic and Phylogenetic Analysis
Source: Mycoses. 2026 Mar 7;69(3):e70161. doi: 10.1111/myc.70161 (PMC12966976; doi:10.1111/myc.70161)

**
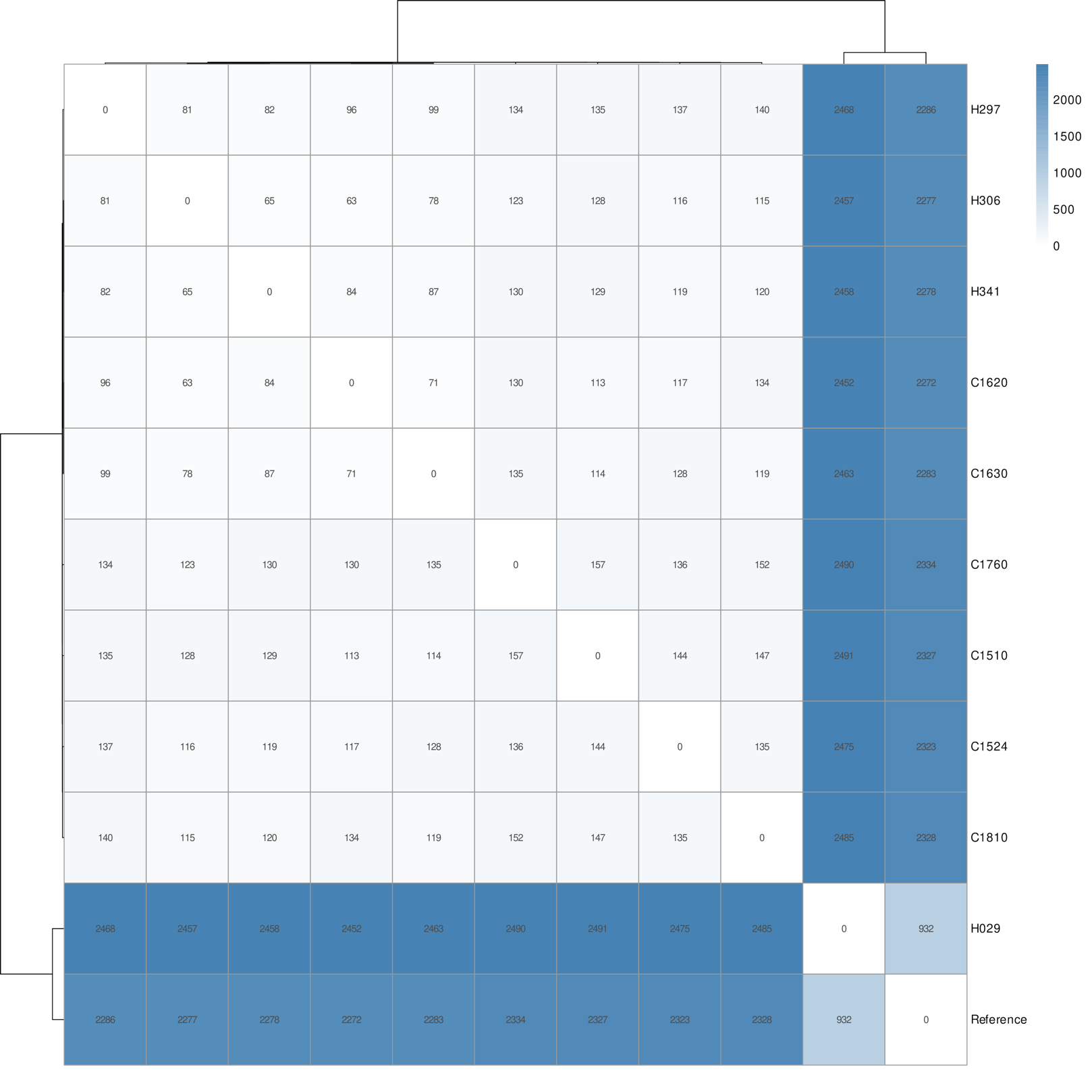
**

**Supplementary Figure 2.** Recombination-masked SNP-distance matrix of the sequenced isolates.

Supplement: Supplementary file 5 — Figure S2:Recombination‐masked SNP‐distance matrix of the sequenced isolates. [file MYC-69-e70161-s005.docx]
